# Mpox (Monkeypox) in Pregnancy: Viral Clade Differences and Their Associations with Varying Obstetrical and Fetal Outcomes

**DOI:** 10.3390/v15081649

**Published:** 2023-07-28

**Authors:** David A. Schwartz, Phillip R. Pittman

**Affiliations:** 1 Perinatal Pathology Consulting, Atlanta, GA 30342, USA; 2 Division of Medicine, U.S. Army Medical Research Institute of Infectious Diseases (USAMRIID), Fort Detrick, MD 21702, USA; phillip.r.pittman.civ@health.mil

**Keywords:** monkeypox, mpox, poxvirus, pregnancy, stillbirth, orthopoxvirus, smallpox, maternal health, clade, monkeypox virus/genetics, pregnancy complications, infectious/epidemiology, maternal-fetal infection, epidemiology, Africa

## Abstract

In African countries where mpox (monkeypox) is endemic, infection is caused by two genetically related clades—Clade I (formerly Congo Basin), and Clade IIa (formerly West Africa), both of which are potentially life-threatening infections. Prior to the 2022–2023 global outbreak, mpox infections among pregnant women caused by Clade I were reported to have a 75% perinatal case fatality rate in the Democratic Republic of Congo, including the only documented case of placental infection and stillbirth from the Congenital Mpox Syndrome, and the Clade IIa mpox infection was associated with stillbirths in Nigeria. The 2022–2023 global mpox outbreak, caused by a genetically distinct strain, Clade IIb, has focused attention on the effects of mpox on pregnant women and fetal outcomes. There have been at least 58 cases of mpox infection occurring in pregnant women during the 2022–2023 outbreak. No confirmed cases of adverse perinatal outcome, including stillbirth, have been reported. The absence of perinatal morbidity and mortality from Clade IIb corresponds to the overall case fatality rate among non-pregnant women of <0.1%, as this clade has been demonstrated to produce a less-severe disease than the mpox Clade I or IIa variants. Thus, there are apparently important differences between mpox clades affecting pregnant women and perinatal outcomes.

## 1. Introduction

Mpox is a major public health problem and an important cause of morbidity and mortality in the endemic countries of Central and West Africa, where it infects many thousands of persons annually. Despite being a potentially lethal zoonotic virus that was discovered over five decades ago, it has attracted little attention from the public health and medical communities. In the endemic African countries, mpox is caused by two genetically related mpox viruses—Clade I (formerly termed Congo Basin clade), and Clade IIa (formerly termed West Africa clade), both of which can produce potentially life-threatening infections. A genetically distinct mpox clade, Clade IIb, was identified as the cause of the 2022–2023 global mpox outbreak [1,2].

There have been scant data accumulated regarding the effect of the mpox virus on pregnant women, fetuses, and neonates and the capability for vertical transmission [3]. Mpox was known to represent a potential threat during pregnancy based upon descriptions of a small number of cases from endemic countries of Africa, but the global mpox outbreak focused attention on the effects of this poxvirus on pregnancies [4]. Mpox has been a neglected tropical disease, and there have been no population-based birth cohort studies of mpox occurring in pregnancy, either prior to or during the current outbreak, and some cases of mpox during pregnancy have had incomplete follow-ups. This communication discusses the most recent information on the consequences of mpox in pregnancy both prior to and during the 2022–2023 global outbreak and investigates whether there may be differences in clinical pregnancy outcomes based upon mpox virus clades.

## 2. Mpox Virus Pathophysiology

The mpox virus is an enveloped, linear, double-stranded DNA virus belonging to the genus Orthopoxvirus and family Poxviridae. Mpox virus is a 200 to 250 nm brick-shaped virus that binds to glycosaminoglycans (GAGs) and mediates endocytosis and viral entry into the host cells [5,6]. The cell entry receptors for mpox have not been confirmed but possibly depend on both viral strain and host cell type. This process could involve cell surface receptors, including heparan sulfate or chondroitin sulfate [7]. As the mpox virus is an enveloped virus, it has been proposed to utilize a classical apoptotic mimicry mechanism for entry into the host cells [6]. Attachment of viral proteins to host cell GAGs is followed by endocytosis and fusion of the virus with the host cell plasma membrane and release of the viral core in the cytoplasm. During the early phase of host cell invasion, approximately 30 min after infection, the early viral genes are transcribed by a viral RNA polymerase, and the core is completely uncoated following the end of early expression. This is followed by the intermediate phase, in which a free viral genome expresses intermediate genes in the cytoplasm, and genomic DNA replication occurs. The late phase of viral replication is characterized by expression of late genes and production of all viral structural proteins, approximately 140 min to 48 h following infection. Assembly of mpox progeny occurs in the cytoplasm, first producing an immature spherical particle that subsequently matures into a brick-shaped intracellular single-membrane mature virion (IMV) [5,6,7]. This IMV can be released following host cell lysis. In addition, the IMV can acquire a double-membrane coat from translocation to Golgi bodies, resulting in virus with a three-layer membrane that is antigenically distinct, and which can bud as an external enveloped virion [5,6,7]. Completion of the mpox virus cycle is dependent on two multi-subunit complexes. The conserved oligomeric Golgi complex is necessary to maintain Golgi structure and intra-Golgi traffic regulation. The Golgi-associated retrograde protein is responsible for retrograde endosomal transport [5,7,8,9].

## 3. Mpox Clades and Differences in Pathogenicity

Following the initial discovery of the mpox virus in a non-human primate in 1958 [10] and the first case of human infection in 1970 [11], two genetically and geographically distinct strains of the virus were recognized [12,13,14]. The Congo Basin or Central African strain was endemic in the Democratic Republic of the Congo and the Central African Republic, and the West African strain was endemic in Nigeria, Cameroon, Liberia, and Sierra Leone [15]. In order to avoid stigmatization and discrimination, the World Health Organization convened an expert committee that suggested renaming the virus. The Congo Basin strain was renamed Clade I, and the West African strain became Clade II. Clade II included two phylogenetically distinct subclades, Clade IIa and Clade IIb [16,17]. Clade I and Clade II demonstrate pronounced genetic differences, having almost twice the divergence as that between subclades IIa and IIb [16]. It appears phylogenetically that both subclades include genomes from the 1960s and 1970s, and it is probable that they evolved separately from a recent common ancestor dating back hundreds of years. It is clear that neither subclade IIa nor IIb are descended from one another [12,17].

There are significant differences in the pathogenicity of these two strains. Clade I (Congo Basin) has a case fatality rate that varies between 10% to 15% in all non-vaccinated individuals and 15% in young children, with known human-to-human transmission [5,18,19,20]. In contrast, Clade IIa (West Africa) is associated with a milder form of the disease, less transmissibility, and a case fatality rate varying between 1% to 6% depending on the study, as well as with less human-to-human transmission than Clade I [5,19,20]. A greater pathogenicity of Clade I compared with Clade IIa is also seen in experimentally infected non-human primates [21,22].

The mpox virus has a large genome consisting of approximately 200 kilobase pairs that encode approximately 190 proteins for use in the creation of new viral particles as well as modulating host cell processes. Clades I and IIa have a 0.55% to 0.56% nucleotide difference, primarily occurring in those regions of the genome encoding for a significant virulence gene, and that likely accounts for differences in clinical severity [21,23,24]. Analysis of the genomes of the two strains have shown that Clade I is predicted to possess 173 functional unique genes, and Clade IIa is predicted to have 171 unique genes. Both African clades share 170 orthologs, and from the protein level are approximately 99.4% identical [21].

Differences in the pathogenicity of Clade I and Clade IIa have been suggested to result from differences in specific gene orthologs. These include *COP-C3L* (an inhibitor of complement enzymes), *BR-209* (IL-1β binding protein), and *BR-203* (virulence protein) [5,12,21]. The D14R is another gene that has an important role in virulence differences between Clades I and IIa—it functions as an inhibitor of complement-binding protein (MOPICE), an important anti-inflammatory factor that is absent from the Clade IIa [21,25,26]. In addition to these, there are additional genes that are candidates for clade-specific differences in pathogenicity.

## 4. Pregnancy with Clade I Mpox Virus Infection

The overwhelming majority of mpox infections have occurred in the Democratic Republic of Congo (DR Congo). The first recognized case of human mpox infection was reported in 1970 in the DR Congo in a 9-month-old boy who was the only member of his family without a smallpox vaccination [27]. In the DR Congo, between 1991 and 1998, there were 511 cases reported [27], with some estimates of greater than 2000 suspected cases of mpox virus infection occurring annually [28]. Recent surveillance data from DR Congo have demonstrated a steadily increasing number of suspected mpox cases that rose from less than 500 cases in 2001 to greater than 2500 in 2018 [19,29]. Between January and September 2020, there were 4594 suspected mpox infections [30]. Parallel to this has been an increase in the median age of affected persons [19].

**Figure 1:**
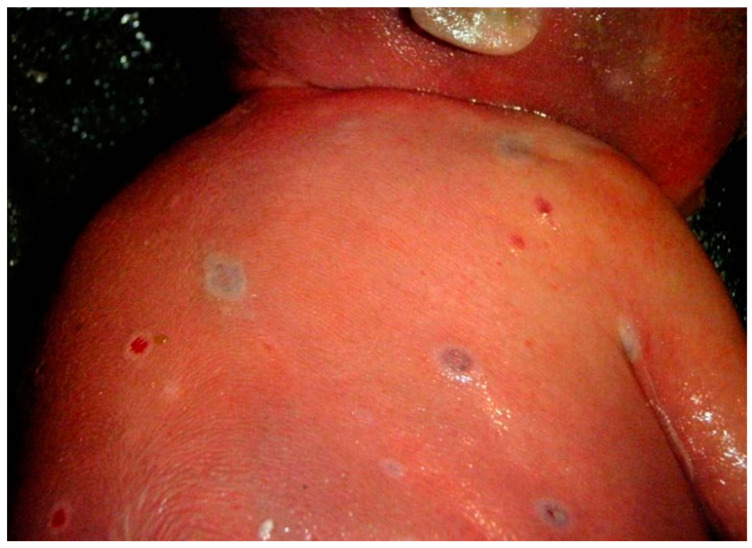
Photograph of mpox skin lesions on the shoulder and back of a stillborn fetus with Congenital Mpox Syndrome taken at the time of autopsy in 2008 in the Democratic Republic of Congo. The fetus became infected following transplacental transmission of Clade I mpox virus.

In order to investigate the natural history of mpox in an endemic region, the Kole Human Monkeypox Infection Study was performed in the Sankuru Province of DR Congo from March 2007 to July 2011 [31,32]. A cohort of 222 symptomatic patients, of whom 36% were female, were enrolled at the General Hospital of Kole located in a remote town in a region of tropical rainforest where mpox is endemic. There were four women identified in this cohort having PCR-confirmed mpox while pregnant, among whom three (75%) experienced a fetal demise. Case #1 occurred in a woman who developed mpox infection at six weeks of gestation and 24 days later had a miscarriage. She had moderate mpox disease, consisting of fever and 76 skin lesions. Case #2 was a woman who developed fever at 6 to 7 weeks of gestation; the disease progressed to severe mpox with 1335 skin lesions. A miscarriage occurred 14 days after the onset of fever. Case #3 occurred in a woman who became febrile at 14 weeks gestation, developed mild mpox with 16 skin lesions, and delivered a liveborn, full-term, uninfected newborn. Case #4 was a pregnant woman who became febrile at 18 weeks gestation and developed a moderate mpox infection with 113 skin lesions. She had confirmed mpox viremia that rapidly increased from 102 to 106 copies/mL with cessation of fetal movement. Twenty-one days after the onset of fever, a stillborn fetus was delivered at 21 weeks gestation [31,32,33]. A specimen taken for PCR at the time of membrane rupture by transcutaneous amniocentesis was positive for mpox virus at the level of 2.6 × 10^7^ genome copies/mL. Additional samples were positive for mpox virus by PCR, including fetal blood from the umbilical vein at 2.5 × 10^7^ genome copies/mL, fetal tissue from autopsy at 1.7 × 10^7^ mpox virus genome copies/mL, sterile peritoneal fluid at a level of 1.6 × 10^3^ genome copies/mL, and placental tissue at a level of 2.4 × 10^7^ copies/mL. The placenta demonstrated mpox virus positivity in villous stromal cells consistent with Hofbauer cells by immunohistochemistry and antibodies to the vaccinia virus. Autopsy examination of the stillborn fetus demonstrated numerous diffuse cutaneous maculopapular lesions involving the chest, head, abdomen, back, and shoulders (Figure 1). The extremities also had mpox skin lesions that involved the palms and soles of the hands and feet. There was hydrops fetalis present, and internal examination revealed prominent hepatomegaly and peritoneal effusions. These findings represented Congenital Mpox Syndrome [34]. The Kole Human Monkeypox Infection Study demonstrated the risks of having mpox infection (Clade I) during pregnancy, as there was a perinatal fatality rate of 75% among gravid participants, with only one of the four pregnant women having mild disease and delivering a full-term healthy infant.

In addition to these four cases, a case of suspected congenital mpox infection occurred in DR Congo in the 1980s, in which a pregnant women developed a poxvirus-like rash and was subsequently confirmed to have mpox. She delivered a liveborn 24 weeks gestation infant having a generalized skin rash suggestive of mpox disease. The child died 6 weeks later of malnutrition [35,36].

## 5. Pregnancy with Clade IIa Mpox Virus Infection

There are little data available on infections with mpox Clade IIa in pregnant women from the endemic West African countries. There were two reports of mpox occurring during pregnancy from the 2017–2018 mpox outbreak in Nigeria [37,38]. In one case, a pregnant woman with a clinical mpox infection had a spontaneous abortion at 26 weeks gestation; there was no description of the fetus or laboratory testing results [37]. The second case of mpox in pregnancy during the Nigerian outbreak was described by Ogoina et al. [38]. This pregnant woman had mpox infection and a spontaneous preterm rupture of membranes together with a spontaneous abortion at 16 weeks gestation, again with no results of laboratory testing. There have been no maternal deaths reported to occur from Clade IIa infection either prior to or during the 2022–2023 mpox outbreak.

## 6. Pregnancy with Clade IIb Mpox Virus Infection (2022–2023 Global Mpox Outbreak)

Following the onset of the global mpox outbreak in May 2022, the virus spread through 112 countries, areas, and territories and resulted in 87,972 laboratory-confirmed cases and 147 deaths as of 24 June 2023 [39]. This outbreak was unique in several ways. It was the first time mpox had spread widely outside of the endemic countries in West and Central Africa. Another unique feature of this outbreak was its epidemiology. Unlike prior mpox outbreaks, the large majority (94%) of persons affected were gay and bisexual men and other men who have sex with men. Among these men, 41% were infected with the human immunodeficiency virus. During this outbreak, people acquired mpox infection during sexual activity from contact with mpox lesions on the skin or mucosal surfaces of partners [40,41,42]. Phylogenetic analysis found that the current mpox outbreak was primarily caused by an offshoot of the Clade II (West African) virus but had a sufficient number of new mutations to be categorized as a new clade, termed Clade IIb [43,44]. The lineage B.1, which includes all mpox virus genomes from the 2022–2023 global outbreak, had likely emerged in Europe in March 2022 [43]. Although the large majority of infections occurred in men during the outbreak, both non-pregnant and pregnant women and neonates were also infected [45,46]. The overall case fatality rate for mpox during the global outbreak was less than 0.1% [23].

The World Health Organization identified 58 female cases with mpox infection who were either pregnant or recently pregnant as of 13 June 2023 [47]. Among them the median age was 28 years. Infection occurred during the 1st trimester in 4 cases, 2nd trimester in 12 cases, and 3rd trimester in 10 cases, with no data available for 30 individuals. In addition, mpox was identified in an individual who was 6 weeks or less postpartum. Thirteen women were known to be hospitalized, but none were identified that required intensive care, and there were no known maternal deaths. In the nine cases where the manner of mpox transmission was known, the most frequent exposure was a sexual encounter (four cases).

In the United States, the first case of a pregnant woman with mpox was reported on 23 July 2022, and, although details were unavailable, the fetus was reported to be uninfected [48,49]. The Centers for Disease Control and Prevention reported 21 pregnant women in the United States with mpox, and 2 women who were infected within 3 weeks following pregnancy during the period from 11 May to 7 November 2022 [50]. These 23 cases represented 3% of 769 mpox virus infections occurring in cisgender women. There were 12 currently or recently pregnant women for whom exposure data were available, among whom 9 reported sexual contact and 3 reported household contact. Data on the trimester of infection were available for 10 women, with 3 occurring during the 1st, 4 during the 2nd, and 3 during the 3rd trimester. Four pregnant women required hospitalization for pain control or cellulitis and were pregnant when discharged. None of the pregnant women needed intensive care, intubation, or had an unplanned delivery. Among 21 women diagnosed with mpox while pregnant, there were 3 outcomes reported, 2 full-term deliveries with no complications, and 1 spontaneous abortion occurring at 11 weeks gestation. No mpox transmission to the neonates was reported. Two pregnant women developed symptoms of mpox symptoms within 3 days following delivery—their newborn babies developed mpox lesions within 1 week of onset of their mother’s symptoms. These two cases almost certainly represented postpartum transmission, with both newborns responding to treatment.

In Brazil, health authorities reported nine cases of mpox among pregnant women by 26 August 2022 [51]. There were four pregnant women in São Paulo, three in Rio de Janeiro, and one each in Minas Gerais and Ceará [52]. Eight of the nine cases were confirmed by PCR testing for mpox virus; the pregnant individual from Ceará tested negative. A São Paolo newspaper reported that an infected mother had passed the transmission phase with mother and baby in stable condition but did not address potential vertical transmission [51,53]. The infected pregnant woman from Minas Gerais had skin lesions; she delivered a healthy baby. She was isolated from her baby following delivery, and there was no evidence of vertical transmission on discharge [51,54]. As of 23 May 2023, there was a total of 22 pregnant women in Brazil with either confirmed or suspected mpox [55]. These pregnant women had a median age of 26 years, with 2 having 1st trimester infection, 11 infected in the 2nd trimester, and 8 infected in the 3rd trimester. Epidemiological features among these women were no different than other confirmed or probable cases of mpox in non-pregnant women. Two women were hospitalized—one for clinical treatment and the other for isolation.

As of 24 February 2023, the Pan American Health Organization reported that among 2278 confirmed cases of mpox infection occurring in women from its member states, 37 were pregnant and 13 required hospitalizations [56]. The distribution of these cases by country was not provided but included cases from the United States and Brazil, as previously discussed.

During the 2022–2023 mpox outbreak, there have been no reports of intrauterine or placental infection or intrauterine transmission of the virus from Europe, Brazil, the United States, or elsewhere; no maternal deaths from mpox have been identified. There have been a few reports of neonates presenting with mpox several days to a week or more after birth, but these cases likely represented postpartum infection. [57,58,59]. Postpartum transmission may be preventable. A case report from Betim, Brazil, described a pregnant woman who developed 15 papular lesions and adenopathy at 37 weeks 3 days gestation, and a skin scraping was positive for mpox virus by PCR [60]. Due to the maternal lesions, the newborn was isolated in a neonatal care unit following delivery; it had no skin-to-skin contact with the mother, and breastfeeding was contraindicated. The baby remained asymptomatic and PCR negative for 21 days of follow up.

## 7. Discussion

As the first mpox virus human infection was identified in the DR Congo in 1970, infections have been almost exclusively limited to the endemic countries of Central and West Africa, with the DR Congo having the large majority of human infections. In addition, there have been reports of travel-related imported mpox in persons in Singapore [61], Great Britain [62], United States [63], and Israel [64]. Unfortunately, despite many thousands of suspected and confirmed infections occurring every year in the endemic countries for decades, mpox has remained a neglected tropical disease with relatively little research conducted on its epidemiology, pathophysiology, and treatment. Transmission of the mpox virus can occur through several routes. Direct mpox transmission can occur via scratches or bites from infected animals, contact with infected body fluids, or viral contamination of raw meat. Indirect mechanisms of transmission include contact with contaminated clothing, bedding, or surfaces [65,66]. Although rare cases of human-to-human transmission were reported prior to 2022 from Nigeria and the United Kingdom, these have been the major mechanisms of infection during the 2022–2023 global mpox outbreak, which predominantly affected gay and bisexual men [40,41,42].

The number of reported cases of mpox in DR Congo and other endemic African countries, including infections occurring during pregnancy, is almost certainly underestimated because of the difficulty in obtaining laboratory confirmation, social inequality, paucity of local diagnostic facilities, affected persons living in remote rural settings, and challenges from armed conflict, civil unrest, and insufficient infrastructure, including poor roads and the current health delivery system. Compounding these issues is a historical record of inconsistency in the criteria for diagnosing mpox, as the World Health Organization did not release standardized diagnostic criteria and case reporting forms until many months after the onset of the 2022–2023 global mpox outbreak [67,68]. Even following their release, there remain inconsistencies in what is considered to be a confirmed case versus a suspected case due to technical and logistical limitations and discrepancies in molecular pathology testing [68,69]. In assessing the prevalence of mpox infections in obstetric and pediatric populations, these challenges are potentially exacerbated by the fact that current diagnostic criteria do not specifically address testing among pregnant women and children, where they may exist specific immunological differences. In Nigeria, the number of mpox infections through 2021 is likely to be under-reported because much of the Nigerian population has been avoiding healthcare facilities due to fear of contracting COVID-19 [70]. These problems have made the surveillance, scientific investigation, and clinical follow-up of mpox infections challenging.

Previous studies on the clinical outcomes of pregnant women infected with smallpox, another member of the Poxviridae family, demonstrated a high mortality rate for the mother, fetus, and newborn prior to its eradication [3,34]. The case fatality during pregnancy was variable depending on the clinical type of smallpox infection, with an overall mortality rate of 34.3%. Fatality rates were lower in cases of variola minor infection and in previously vaccinated mothers, higher (approximately 40%) in 3rd trimester infections, and almost uniformly fatal when a pregnant woman developed hemorrhagic smallpox [34].

Unlike the situation with smallpox, there are currently no thorough studies available on clinical outcomes of mpox infection in pregnancy. Using the sporadic reports in the literature of individual and small numbers of cases, some from resource-poor countries, makes it difficult to evaluate the risks of adverse maternal and perinatal outcomes. Within these limitations, this communication examined reported cases of mpox in pregnant women with particular attention to their geographic distribution in order to evaluate potential differences in clinical maternal and perinatal outcomes based upon the clade type of the mpox virus.

There appear to be differences in the risk for adverse perinatal outcomes depending upon mpox clades. Based upon published literature, mpox Clade I has a 75% perinatal fatality rate. Although this figure was generated from a single study in DR Congo, the four pregnant women in that investigation were not pre-selected and, with the exception of concomitant malaria in one woman, had no significant obstetrical co-morbidities. Their pregnancies culminated in two miscarriages, one stillborn fetus with confirmed infection and Congenital Mpox Syndrome, and one live birth [31,32,34]. This should not be surprising, as mpox Clade I has the highest case fatality rate of all mpox clades among non-pregnant individuals, between 10 and 15%. The data from Nigeria of women with mpox Clade IIa infection during pregnancy are limited to two individual case reports [37,38], both of which culminated in fetal deaths. D’Antonio et al. [71] performed a metanalysis of all reported cases from Africa of mpox occurring in pregnancy and found that miscarriage occurred in 39% (95% confidence interval (CI), 0–89%) of pregnancies, and intrauterine fetal demise occurred in 23% (95% CI, 0–74%). The overall fetal and perinatal death rate was 77% (95% CI, 26–100%). Fetal loss was 67% (95% CI, 9–99%) in 1st trimester and 82% (95% CI, 17–100%) in 2nd trimester infections. Only 23% (95% CI, 0–74%) of fetuses survived to birth. The analysis of vertical mpox transmission was hindered because some of the fetal demises occurred in the early 2nd trimester, and no confirmatory analysis was performed on the fetal tissues. The exception was the fetal demise at 21 weeks gestation with Congenital Mpox Syndrome from DR Congo that had mpox virus confirmed by PCR and exhibited placental infection [32,33,34].

In contrast to these reports, during the 2022–2023 global mpox outbreak caused by the Clade IIb mpox virus, there have been at least 58 cases, and very probably more, of infection in pregnant women with no confirmed cases of fetal infection or intrauterine transmission. Phylogenetic analysis found that the current mpox outbreak was primarily caused by an offshoot of the Clade II (West African) virus but had a sufficient number of new mutations to be categorized as a new clade, termed Clade IIb, which have been associated with maternal mpox infection. The absence of perinatal morbidity and mortality from Clade IIb corresponds to the overall case fatality rate among non-pregnant women of <0.1%, as this clade has been demonstrated to produce less severe disease than mpox Clade I or IIa variants. While the dramatic differences in perinatal mortality between infections among pregnant women with mpox Clades I, IIa, and IIb may be due to most cases of clade IIb occurring in the United States and Europe with better and faster diagnostics and patient care services, we do not believe this to be the case. During the Kole mpox study in DRC, the pregnant participants received thorough and regular prenatal care that included repeated laboratory testing, nutritional support, vitamins and medications, unlimited access to physicians and hospital care, and treatment of mpox infection [32]. Despite these efforts, three of the four fetuses from pregnant mothers having an mpox infection died. This apparent difference between perinatal mortality between mpox clades may be analogous to the recent situation during the COVID-19 pandemic, in which there have been differences in the risks for miscarriage and stillbirth associated with differing variants of SARS-CoV-2 [72,73].

Unfortunately, the lack of attention and research funding directed toward mpox infection by both the public health and medical communities has resulted in many lost years of investigation that would have improved our understanding of methods for the prevention, surveillance, investigation, and clinical management of mpox. This is especially true with mpox in pregnancy, where our information is limited [3,34,74]. There are many unknowns: are pregnant women are more susceptible to mpox virus? Is mpox infection more severe when it occurs during pregnancy? What is the relationship between mpox clades, development of maternal viremia and adverse perinatal outcomes? What are the features of mpox infection of the placenta? In future clinical trials of potential drugs and vaccines for mpox, pregnant women should be enrolled as participants, as has recently occurred with other emerging viral diseases [75,76,77]. It is of great importance that more studies are performed to understand the epidemiological features of risk, vertical transmission, and clinical consequences of mpox infection occurring in pregnancy.

## Data Availability

Data are available upon reasonable request from the corresponding author.
